# Supplementary figures and images for: CRISPR gRNA phenotypic screening in zebrafish reveals pro-regenerative genes in spinal cord injury
Source: PLoS Genet. 2021 Apr 29;17(4):e1009515. doi: 10.1371/journal.pgen.1009515 (PMC8084196; doi:10.1371/journal.pgen.1009515)

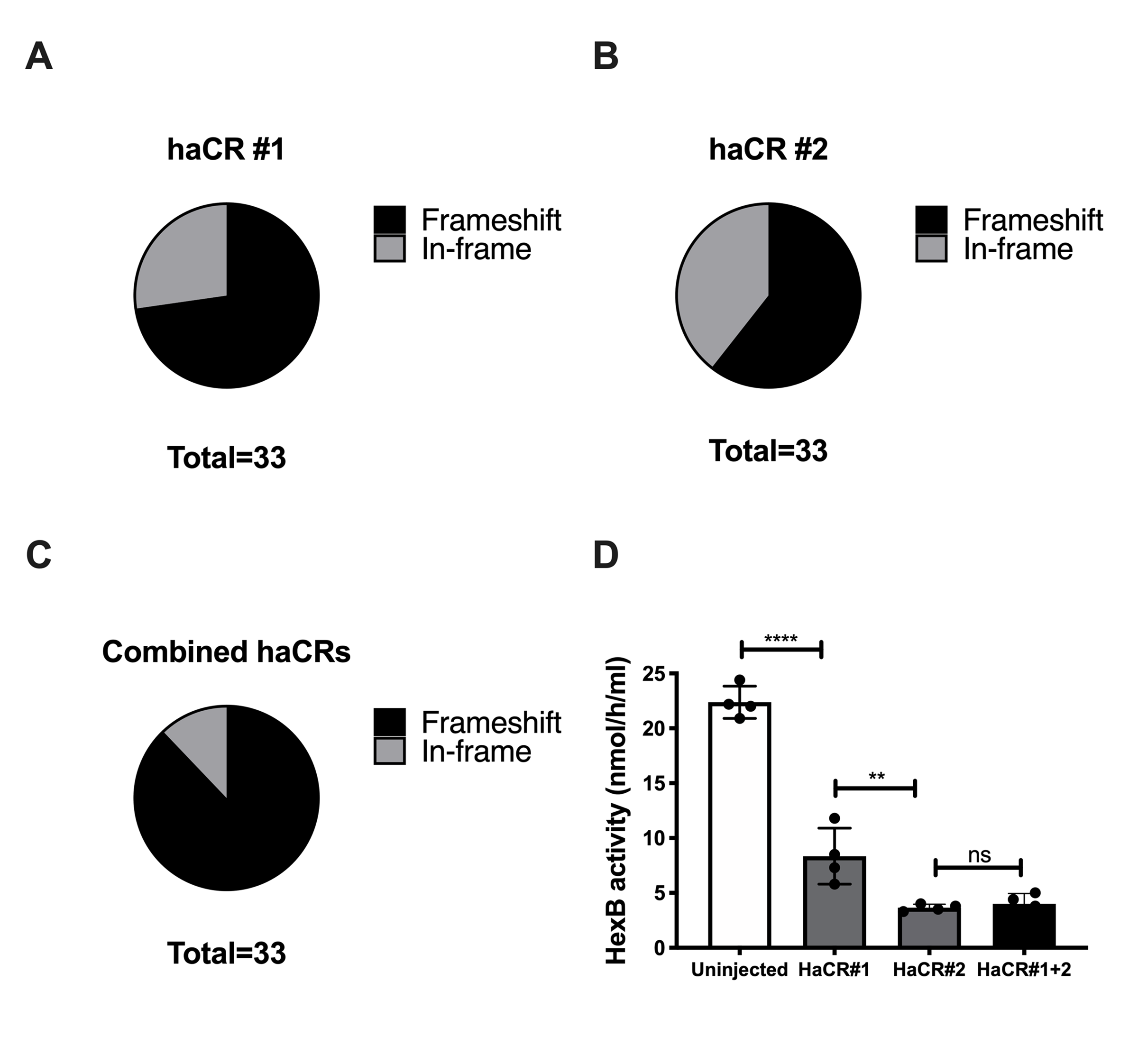

Supplement: S1 Fig — A-C: Direct sequencing of mutant alleles in embryos injected with two haCRs per gene demonstrates induction of frameshift frequencies of 72% (A; haCr #1) and 60% (B; haCR #2), when either sCrRNA is analysed individually. This rises to 87% when frame-shift frequencies are combined (C). D: At the protein level, dual haCR injection against hexb reduces enzyme activity by 80% in vivo (ANOVA with Tukey post-test; p < 0.0001). Single haCRs also reduce enzyme activity (p < 0.0001 for both). haCR#2 is more efficient than haCR#1 (p = 0.0051). Error bars represent SEM. (TIF) [file pgen.1009515.s001.tif]

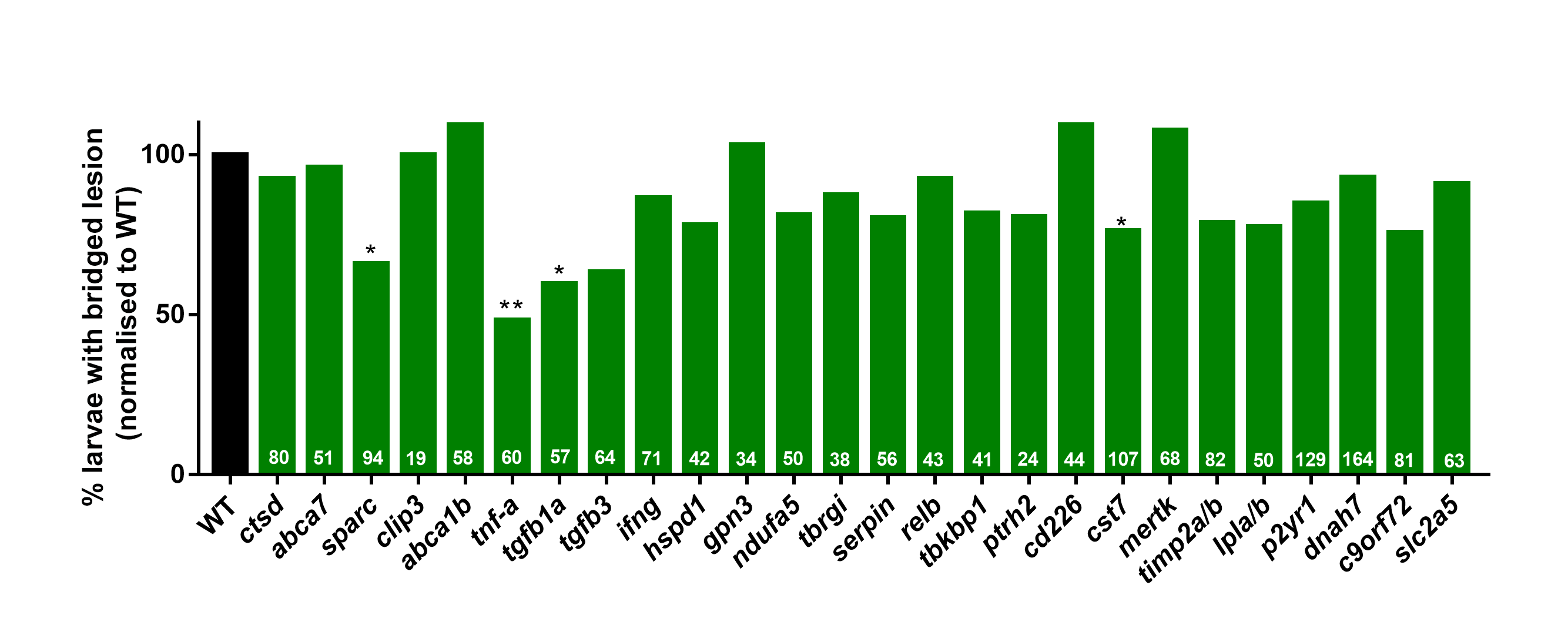

Supplement: S2 Fig — Significant reductions in bridging were detected after acute injection of haCRs for sparc (p = 0.0424), tnfa (p = 0.0068), tgfb1a (p = 0.0225) and cst7 (p = 0.0418). Fisher’s Exact test, p<0.05. (TIF) [file pgen.1009515.s002.tif]

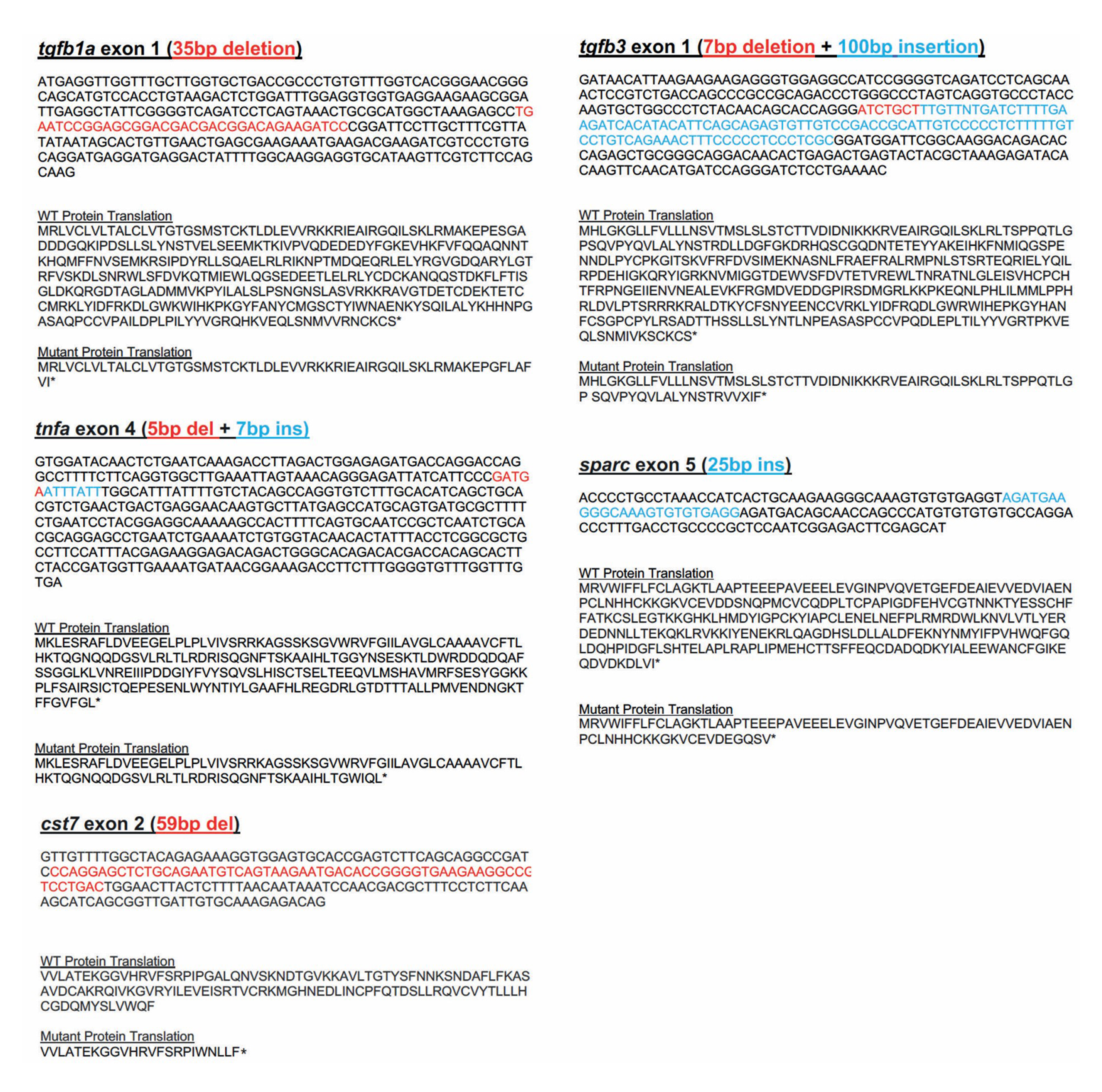

Supplement: S3 Fig — Deletions are shown in red and insertions are shown in blue. All stable mutations produce frameshifts (cst7, tnfa, tgfb1a and sparc) and premature stop codons, with the exception of the mutation in the tgfb3 gene. The latter contains an in-frame indel in which the large quantity of inserted material contains a nonsense mutation. (TIF) [file pgen.1009515.s003.tif]

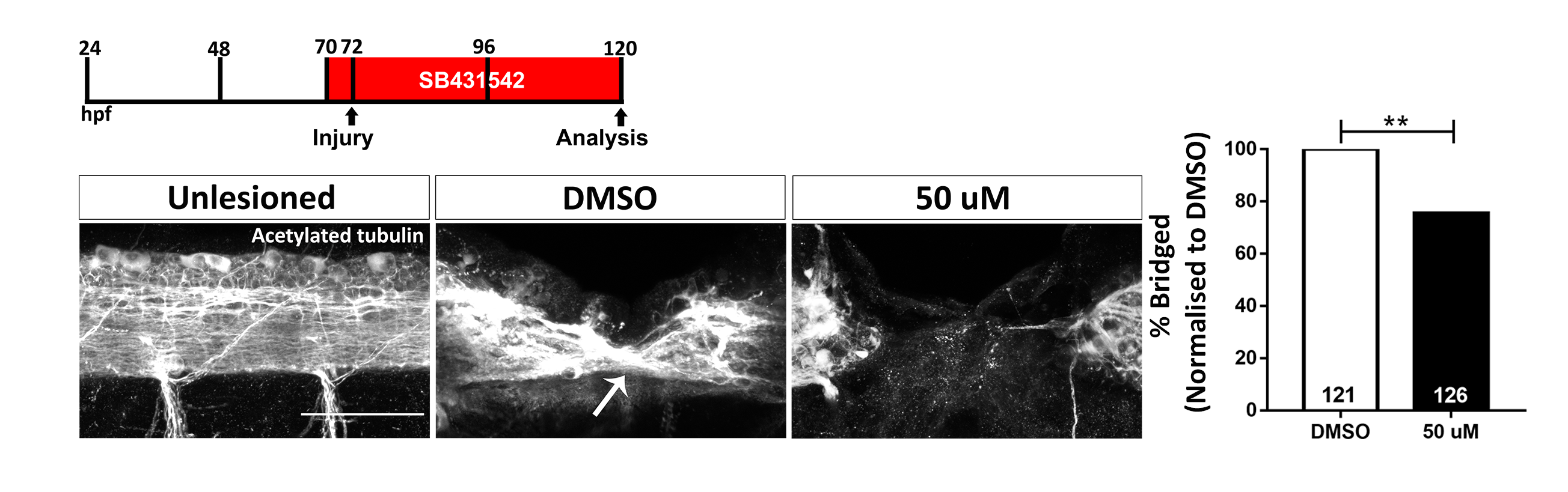

Supplement: S4 Fig — Experimental timeline, lateral views of embryos and quantification are shown. Fisher’s Exact test, p<0.05. The white arrow indicates the axonal bridge. Scale bar 50 μm. (TIF) [file pgen.1009515.s004.tif]

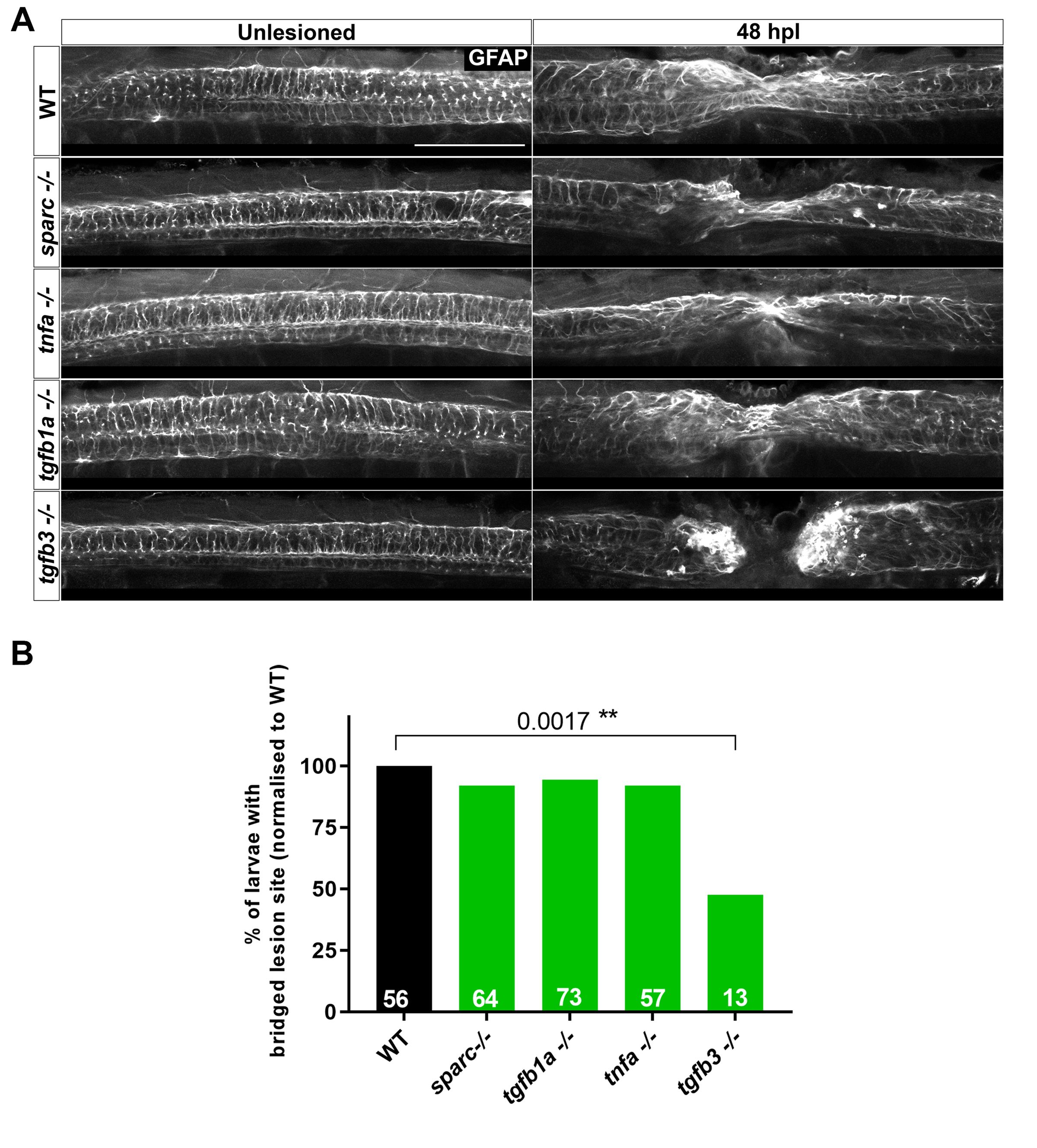

Supplement: S5 Fig — Lateral views of whole-mounted larvae at 5 dpf are shown; rostral is left, dorsal is up. A: Gfap immunohistochemistry shows longitudinal processes over the injury site (centred in right column) for wildtype (WT), sparc, tnfa, and tgfb1a mutants, but not for tgfb3 mutants at 48 hours post-injury (hpf). B: Quantification of the phenotypes shows a significant reduction in the proportion of larvae with glial bridging only for tgfb3 mutants (Fisher’s Exact test). (TIF) [file pgen.1009515.s005.tif]

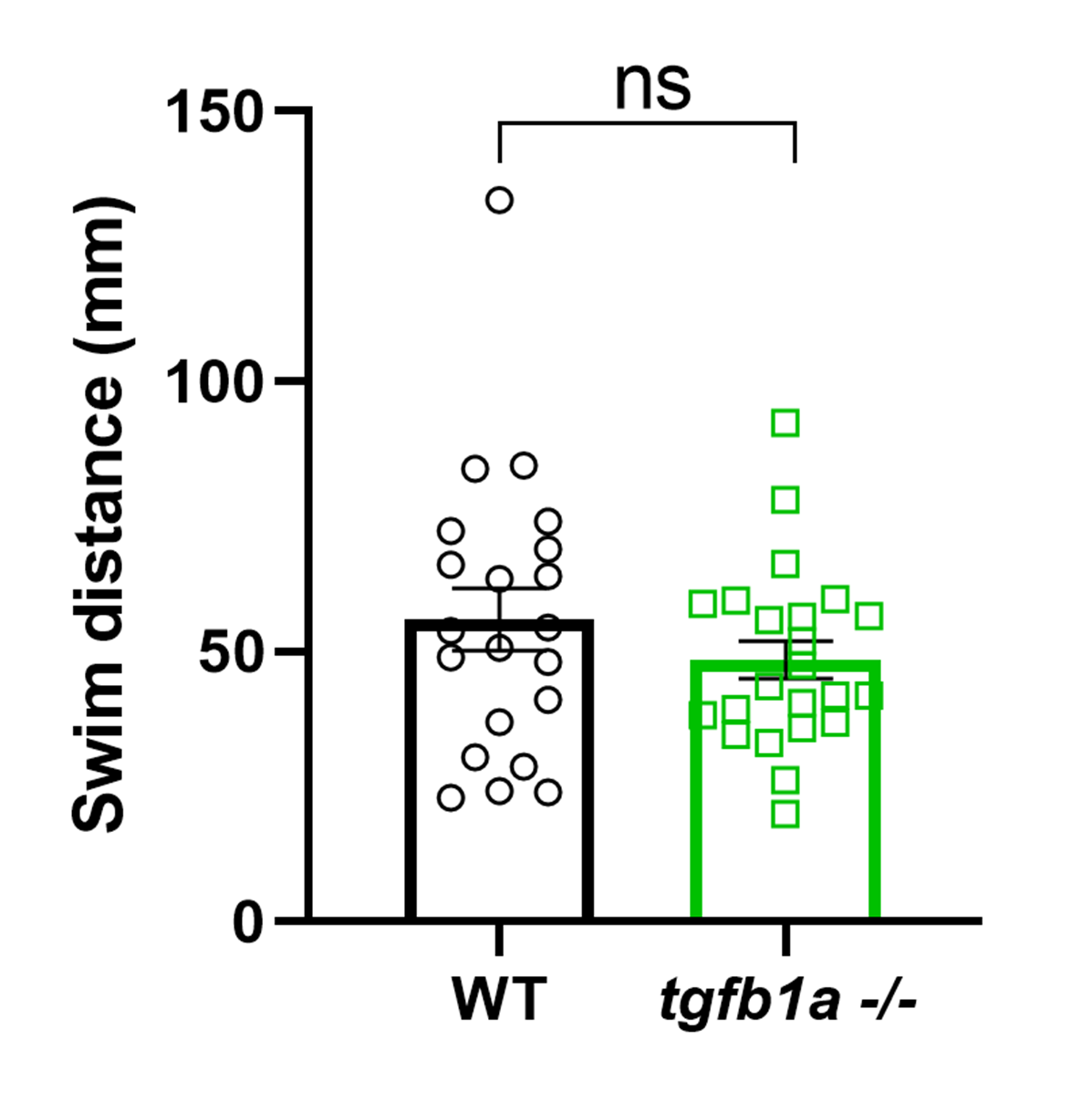

Supplement: S6 Fig — No change is observed in the distance that tgfb1a mutants swim after a touch evoked stimulus (Unpaired t test, p = 0.2590). (TIF) [file pgen.1009515.s006.tif]
