# Supplementary material for: CRISPR gRNA phenotypic screening in zebrafish reveals pro-regenerative genes in spinal cord injury
Source: PLoS Genet. 2021 Apr 29;17(4):e1009515. doi: 10.1371/journal.pgen.1009515 (PMC8084196; doi:10.1371/journal.pgen.1009515)
Supplement: Attachment — Submitted filename: Plos_Genet_rebuttal_090321.docx [file pgen.1009515.s009.docx]

**Comments to the Authors:
Please note here if the review is uploaded as an attachment.**

Reviewer #1: In this manuscript the authors describe a method for more selecting more highly active GRISPR guides and functionally test them in the context of larval zebrafish regeneration. The method for selecting and testing the guides seems robust and applicable to organisms with a sequenced and very well annotated genome. The manuscript is well written but in some cases assumes the reader is very familiar with previous research in the field, the manuscript should be revised to ensure a reader can understand the manuscript without having to go read other papers. Specific points:

1. The authors select a list of genes to test with their new guide design method which they say are “macrophage related genes based on previous research”. Table S1 should be revised to include specific information about these genes, reference papers, have they been previously knocked out, what is the phenotype, what method was used, morpholinos, mutants, talens etc.

That is a helpful suggestion and we have added the requested information in the new Table S2.

2. Information should be given in the text or Table S1 as to where these genes are normally expressed in development and is there an embryonic phenotype from knocking them out in the embryo?

We have now included this information in Table S2.

3. Are the genes they selected normally up-regulated after spinal cord injury, is this why they were selected?

Genes are either upregulated or highly expressed in injury contexts. We have added this information to Table S2.

4. The authors use an axon regeneration assay using the Tubb:DsREd transgenic line to assay axon regeneration. Previous work from others in the field have shown that the glial cells must first migrate and form a glial bridge and then axon regeneration begins. It would be interesting to know if there are also defects in the formation of the glial bridge in mutants where axon regeneration is impaired.

We have addressed this question experimentally for the mutants with pronounced axon phenotypes and we find impaired crossing only for tgfb3. We have added the data as new Fig. S5 and discuss this in the context of the relationship between glia and axons on p. 16:” Previous work has suggested that bridges of glial processes across the injury site may form a substrate for axons to cross the injury site in adult zebrafish (Goldshmit et al., 2012; Mokalled et al., 2016). However, time-lapse observation in larvae has shown that axons often grow independently of glial processes and also cross the injury site when glial processes are ablated (Wehner et al., 2017). Here we observe that most mutants do not show glial phenotypes and find that the *tgfb3* mutant exhibits a strong glial phenotype, but a relatively mild axonal phenotype. This suggests differences in glial and axonal crossing mechanisms of the injury site and that our screen is sensitive to genes that are necessary for axonal regrowth.”

5. Five genes which gave phenotypes were raised as stable mutants. Again it would be helpful to know where/which cell types these genes are normally expressed in after spinal cord injury. This might shed more light on why KO of tgfb1a and tgfb3 do not result in any change in the number of macrophages at the injury site, when increased number of neutrophils are observed, do they stay at the injury site for longer than in WT animals, the same with animals where an increase in macrophages in seen?

We agree that this is a fascinating line for future research. Unfortunately, in the context of establishing a screening assay, we feel that such additional analyses would be out of scope for this study. Instead, we added known gene expression of the genes in question, mainly in macrophages, to Table S2 and we state in the manuscript on p. 13:” Higher neutrophil counts are likely a consequence of an impaired ability of macrophages to control neutrophil numbers, e.g. by inducing reverse migration of these (Tauzin et al., 2014).”

6. Overall the assay of axon regeneration and effect on immune cells are quantified at very early timepoints, even in young larvae axon regeneration takes more than 48hrs, previous work from Briona and Dorsky report axon regeneration to take up to 9 days in 4 day old larvae, while work from the Saude group reports up to 6dpl. These groups and others work on spinal cord regeneration in zebrafish, it would be good to see references to other people work in the field in this manuscript.

This is a helpful suggestion. Those colleagues use slightly different regeneration paradigms. We see asymptotic, almost full functional recovery already at 48 hours after injury at 3 days post-fertilisation, so our chosen time points for anatomical analysis are also behaviourally relevant (see below image; wt = wildtype; from (Tsarouchas et al., 2018)). We have added this information and discuss it in the context of the observations by others on p. 10: ”While we have shown recovery of swimming capacity in this paradigm by 48 hpf (Tsarouchas et al., 2018; Wehner et al., 2017), full regeneration might take longer, as observed in other larval lesion paradigms (Chapela et al., 2019; Vasudevan et al., 2020).” We cite additional literature here and in other places.

Reviewer #2: This study screened for macrophage genes implicated in spinal cord injury in zebrafish larvae. Recently reported high efficiency CRISPR methods were used. 30 macrophage genes were targeted. Phenotypes were scored for the presence or absence of an axon reporter bridge at the lesion site. Phenotypes were observed and confirmed in stable lines for tgfb1a tgfb3 tnfa and sparc. The study proposes that tgfb1a deletion leads to a prolonged immune response that inhibits regeneration after injury.

1- One concern is that the described screening methods report low targeting efficiencies relative to previously published studies in the field. In this study, 44% of all tested RNAs were high efficiency. This is in contrast to findings from Hoshijima et al, which convincingly reported bi-allelic indel mutations in almost all cells of the developing embryos, a result that has been recapitulated by numerous other labs. The authors do not discuss or address the reasons behind limited targeting efficiency, and instead propose pre-screening for high efficiency guide RNA to overcome this limitation.

We thank the reviewer for highlighting this crucial point. We might have made it insufficiently clear how we go beyond the Hoshijima paper. In agreement with Hoshijima et al, we find that somatic mutations are highly efficient for specific synthetic guides. Hoshijima et al do not address whether sCrRNAs are highly active in general, they analyse a small number of *pre-selected* highly active sCrRNAs. Importantly, we demonstrate for the first time across 350 guides that activity of guides needs to be individually assessed. We make this more explicit in the manuscript on p. 18f:” Although very high somatic activity of specific sCrRNAs has been demonstrated (Hoshijima et al., 2019), only an unbiased testing of large numbers of sCrNRAs could reveal variability”

a. The design strategy was limited to targeting enzyme recognition sites that were later used for restriction fragment length polymorphism (RFLP) genotyping. Is this limitation likely to limit targeting efficiency? Especially considering that enzyme-independent assays are now available to accurately quantify the rate of mutagenesis.

We have tested a number of variable recognition sites using a variety of enzymes, but the variability in activity of sCrRNAs persists. Enzyme recognition sites are therefore unlikely to explain the different targeting efficiencies. We expand the discussion of methodological limitations in the discussion on p. 18: “It is unlikely that the enzyme recognition sites chosen for our RFLP-based sCrRNA activity are particularly resistant to CRISPR manipulations, because we used varied enzymes with unrelated sequences, yet variability in activity remained. Nevertheless, other highly efficient methods to determine sCrRNA activity, such as deep sequencing are available (Anzalone et al., 2019). Chemical modification of the RNA oligos are also unlikely to boost efficacy (Hoshijima et al., 2019). Finally, experimental variations, such as injection location and vehicle composition are unlikely to decisively influence effectiveness of sCrRNAs, as described by others (Wu et al., 2018) and also reflecting our own experience. Hence, while we cannot exclude that optimisation of experimental protocols could increase haCR frequency, the above considerations suggest sCrRNA activity may be intrinsically variable. Without knowing the complex reasons for these differences, pre-screening presents a viable work-around for screening purposes.”

b. Compensatory mechanisms associated with targeting early exons have been described in great detail in the field but are not addressed in this study. Mutations with premature stop codons are not always likely to abrogate gene function as stated in the manuscript. Added to this concern is that all 4 phenotypes reported in this study were attributed to genes in which functional domains rather than 5’ exons were targeting.

This is an interesting observation. We address this in the manuscript on p.17: “We cannot exclude false negative findings resulting from functional protein still produced despite premature STOP codons being introduced. However, this is the exception rather than the rule, and generally only occurs when splice sites have been mutated (Anderson et al., 2017). A two-haCR approach will likely limit false negative findings.”

c. A number of genes that are essential for macrophage function do not show phenotypes. Is this due to targeting strategies? Do stable p2yr1 mutants shown normal recovery for example?

We agree with the reviewer that the analysis of more stable mutants could be interesting. However, for a screening approach, we cannot follow up negative findings. While some genes may be essential for specific macrophage functions, these functions may not affect the specific read-out for our screen. To make this clearer now write on p. 18: ”It is also important to state that a negative result in the highly targeted read-out for our screening assay does not mean that targeted genes are not important for other immune functions.”

d. Is it possible that the source of synthetic RNA is a contributing factor. Unclear whether the RNAs used here are chemically modified to enhance their resistance to degradation the way Alt-R-modified RNAs were shown to be.

We apologize for omitting the information that our sCrRNAs are unmodified. This is based on observations by others that there is no clear impact of these modifications. We write in the text on p. 18 now: “Lack of chemical modification of the RNA oligos is also unlikely to be responsible for variable efficacy according to findings by others (Hoshijima et al., 2019).”

2- The more exciting and novel aspect of this study is to apply recent CRISPR advances in zebrafish genetics to identify new genes involved in spinal cord injury. Unfortunately, the inflammatory factors tgfb1 and 3 are well characterized in spinal cord injury, and the data presented seems preliminary. For example, this reviewer finds sparc phenotypes intriguing and novel. Including expression and phenotypic data for sparc can add the missing novelty to the study. Keeping in mind that Sparc is also a known regulator of synapse function, it is important to distinguish between possible functions in neurons or macrophages.

We agree that sparc is an intriguing candidate for future analysis, which will contribute to the interest in our screening manuscript. However, because the emphasis is on the screening approach, such analyses go beyond the scope of the present study. We integrate the reviewer’s interesting observation on p. 16: ”However, *sparc* has multiple functions, for example in synapse formation (Jones et al., 2011; Kucukdereli et al., 2011), which will be analysed in future detailed phenotypic analyses of the mutant.”

3. The rationale behind which macrophage-related genes were included is not described in the text. it will be helpful to describe their expression and previous implication in macrophage activation.

We have now included the available information in our new Table S2.

4. Phenotyping was based on scoring an axon reporter line for bridging at the lesion site. This method does not distinguish phenotypes related to degeneration and debris clearing from regeneration phenotypes. The authors have previously reported numerous methods to assess anatomical and behavioral regeneration. We recommend using these assays for in depth characterization of the phenotypes.
As an example, Figure 3 gives a brief overview of the phenotype in tgfb mutants. Quantifications in 3b and 3c suggest that macrophage number were not changed but that tgfb1a mutants have more neutrophils. It is possible this difference is statistical since many more replicates are quantified in 3c.

Unfortunately, in-depth analysis of phenotypes for all mutants would detract from the main point of the manuscript – introduction of a robust screening paradigm – and would go beyond the scope of a single paper. Instead, we have extended our exemplary analysis of the tgfb1a phenotype in terms of motor recovery (new Fig. S6) and rescue by il-1b inhibition (new Fig. 3G).

With regard to macrophage numbers, we do not observe any trend towards a change in numbers. Power analysis indicates that to observe a similar increase as for neutrophils, we already have a power of 0.85 (target power > 0.8). That means that an absence of an effect on macrophage number is highly unlikely to be a consequence of underpowering our study. We have added the power analysis to the legend of Fig. 3: “Theoretical power = 0.85 to see a similar increase as for neutrophils in C”

Reviewer #3: The manuscript by Keatinge et al. describes a very valuable and useful scalable in vivo phenotypic screen based on CRISPR technology using imaging-friendly zebrafish larvae.
The screen was designed for a specific spinal cord injury paradigm, but I would like to emphasize the possibility of using the same screening logic to identify regulators of other biological processes, as long as they are easy to phenotype in medium-large scale.

We strongly agree with the reviewer that it is important to point out the versatility of our approach and we state now in the conclusion: ”We are using a specific injury paradigm as a read-out. However, our sCrRNA approach can be used in a variety of developmental or injury contexts where specific cell types may be transgenically labelled and a simple and robust read-out can be devised or be gleaned from small molecule screens in zebrafish (Lam and Peterson, 2019). This could be done in an automated fashion to further increase throughput (Early et al., 2018).”.

As an important note for the general scientific community, the authors claim that the 4 different in silico prediction tools they used did not have enough prediction power to selected highly efficient guide RNASs. They propose the use of an easy and efficient RLFP-based tool and further show its efficacy in the prediction of guide RNAs.

I have a few points that I would like the authors to address.

\# 1. The screening platform was designed to screen for macrophage-related genes and was successful in identifying potential macrophage players, with potential inflammation modulation effects and with potential positive effects on spinal cord regeneration. I would like to emphasize the word potential because at this stage of the work that's what the identified genes are. I think the authors should lower the tone throughout the manuscript about the involvement of these genes specifically with macrophages (as no co-stainings were done) and with regeneration capacity (as no swimming recovery test of any type was performed, only the presence of tubulin:GFP at the injury site was roughly quantified).

We agree with the reviewer and we have carefully moderated our statements on macrophages throughout the manuscript. In addition, we are now stating more explicitly the reasons for including specific genes in Table S2.

\# 2. In Fig 1A, why do the Uninjected Digested lanes of irak3 gene have only one band? If the embryos were not injected, they should have the restriction site intact and when digested should then produce at least 2 bands. Please clarify.

We thank the reviewer for spotting this mistake. We accidentally over-cropped the image. This is now corrected to show the second band for *irak3*. We show below the full gels for the three indicated examples for reference.

Cst7

Irak3

Acba1a

\# 3. In Fig 2A, I think the representation of axonal bridge at 24 and 48 hpl is misleading, as it is expected that at 48 hpl the axonal bridge at the injury site would be bigger when compared to the one seen at 24 hpl.

This is a helpful comment and we have changed the cartoon accordingly.

\# 4. In Fig 2B, the examples of transgenic larvae shown are at which time point after lesion?
I find bridging a weak and limited item to access in this context. You can find larvae with a lot or very little bridging and these will be included in the same bridged category. In their quantification methodology, a larvae with only on fascicle being continuous between rostral and caudal spinal cord ends will be included in the bridged category. And the question I would like to raise at this point is to what extent can we came assumptions on the regenerative capacity based on only one fascicle crossing the injury site? A swimming test would be essential to produce a stronger conclusion.

We apologize for not having made clearer that the scoring assay for axon regrowth has been established in two previous papers, showing that the percentage of larvae with an axonal bridge correlates directly with the recovery of swim capacity (Wehner et al., 2017). The same is true for bridge thickness measurements (Tsarouchas et al., 2018). In a screen, simple read-outs are essential. We have added this explanation to the manuscript on p. 10: “Determining the percentage of larvae with continuity of axonal labelling across the spinal injury site (called “bridging”) at 24 and 48 hpl is very rapid, a necessity in a screening application. At the same time this previously established scoring method is a sensitive measure to detect changes in the efficiency of axonal regeneration (Tsarouchas et al., 2018; Wehner et al., 2017). For example, we have shown that our scoring method correlates with more complex measurements, such as the thickness of the axon bridge, and with the degree of recovery of swimming function animals show after injury.“

\# 5. In Fig 2C, the y axis should not be % of larvae with bridged lesion because theses values were normalized to the WT control (in which according to values in Fig 2A a represented approx. 80% and not 100% as seen in the 2C graphic.

We thank the reviewer for spotting this mistake and we have corrected the axis labelling accordingly.

\# 6. In Fig 2D, why is there a discrepancy between the bridging % tendency between acute injections and stable mutations? The difference expected would be that in stale mutants the effect would be more pronounced than in the acute mosaic injections, why is this? and did they authors also see the defects at 24 hpl that they saw in the acute injected ones?

This is an important point and we have performed new assessments of the *sparc*  axon bridging phenotype at 48 hpl, which is our standard time point for comparison. The data has been incorporated into the table in Fig. 2D and changes the discussion as indicated in response to point #7.

\# 7. In Fig 2D, the legend says that the sparc mutant was analysed at 24 hpl but the text gives the idea that the result is from 48 hpl. Is the result the same for 24 and 48 hpl? Could the stronger phenotype of the sparc mutants be a result of the earlier timepoint analysed?

Based on the improved comparison indicated in response to point #6, we can now say that mutant phenotypes either match those of acute injection or are weaker. We discuss this on p. 19f: “However, even with our 2-guide approach, we encountered a false positive in *cst7* and two of the other four mutants showed a weaker phenotype than after acute injection of haCRs. Despite CrRNAs having few predicted off-target effects, we cannot exclude that these exaggerated the phenotype. Sensitivity to off-target effects may also vary depending on design and read-out, which will be revealed in future screens. Overall, this highlights that guide number should be kept to a minimum and that validation of novel phenotypes with stable mutants is advised.”

\# 8. The authors when on to characterize in more detail the tgfb1a mutant and propose that Tgfb1a signaling may be part of the mechanism by which macrophages control iL1b levels and thus promote regeneration. To support this hypothesis the authors could simply do a IL1b inhibition rescue experiment similarly to what they already did for the tnfa mutant. And I would argue that it would be fundamental to complement these experiments with swimming recovery tests (tgfb1a mutant and tgfb1a mutant after rescue).

These are excellent suggestions and we have now performed these experiments. Indeed, we can show that blocking il-1b release significantly reduces the axon bridging phenotype in the tgfb1a mutant, indicating that control of il-1b levels by tgfb1a is necessary for axonal bridging. We have added the data as Fig. 3G.

We have also performed a test for swim distance for the tgfb1a mutant, but only found a non-significant reduction in touch-evoked swimming, added as suppl. Fig. S6. There was probably no significant drop in functional recovery, because there is still noticable axon bridging occurring in the mutant and the relationship between axon regrowth and functional recovery is non-linear. We write in the text on p. 14: “We also analysed touch-evoked swimming distance in *tgfb1a* mutants. However, no significant impairment was observed (suppl. Fig. S6). This may be explained by a non-linear relationship between relatively modest anatomical recover already leading to substantial functional recovery after spinal cord injury (Becker et al., 1997)”

\# 9. Regarding the genotyping of the stable mutants used for the phenotypic analysis (Fig 2D), it is not clear in the methods how this was done. Do the authors use individual larvae to extract DNA after doing the injury and then genotype by PCR?

That is the correct. In addition to the Material and Methods section, we now also mention this in the results to make this clearer: “To do this efficiently, we targeted recognition sites for restriction enzymes, such that we could use resistance to enzyme digestion in restriction fragment length polymorphism analysis (RFLP) of individual embryos as a proxy for mutagenesis activity at the target site (Fig. 1A).”

Becker, T., Wullimann, M.F., Becker, C.G., Bernhardt, R.R., and Schachner, M. (1997). Axonal regrowth after spinal cord transection in adult zebrafish. J Comp Neurol *377*, 577-595.

Chapela, D., Sousa, S., Martins, I., Cristovao, A.M., Pinto, P., Corte-Real, S., and Saude, L. (2019). A zebrafish drug screening platform boosts the discovery of novel therapeutics for spinal cord injury in mammals. Sci Rep *9*, 10475.

Early, J.J., Cole, K.L., Williamson, J.M., Swire, M., Kamadurai, H., Muskavitch, M., and Lyons, D.A. (2018). An automated high-resolution in vivo screen in zebrafish to identify chemical regulators of myelination. eLife *7*.

Hoshijima, K., Jurynec, M.J., Klatt Shaw, D., Jacobi, A.M., Behlke, M.A., and Grunwald, D.J. (2019). Highly Efficient CRISPR-Cas9-Based Methods for Generating Deletion Mutations and F0 Embryos that Lack Gene Function in Zebrafish. Dev Cell *51*, 645-657.e644.

Jones, E.V., Bernardinelli, Y., Tse, Y.C., Chierzi, S., Wong, T.P., and Murai, K.K. (2011). Astrocytes control glutamate receptor levels at developing synapses through SPARC-beta-integrin interactions. J Neurosci *31*, 4154-4165.

Kucukdereli, H., Allen, N.J., Lee, A.T., Feng, A., Ozlu, M.I., Conatser, L.M., Chakraborty, C., Workman, G., Weaver, M., Sage, E.H.*, et al.* (2011). Control of excitatory CNS synaptogenesis by astrocyte-secreted proteins Hevin and SPARC. Proc Natl Acad Sci U S A *108*, E440-449.

Lam, P.Y., and Peterson, R.T. (2019). Developing zebrafish disease models for in vivo small molecule screens. Curr Opin Chem Biol *50*, 37-44.

Tauzin, S., Starnes, T.W., Becker, F.B., Lam, P.Y., and Huttenlocher, A. (2014). Redox and Src family kinase signaling control leukocyte wound attraction and neutrophil reverse migration. J Cell Biol *207*, 589-598.

Tsarouchas, T.M., Wehner, D., Cavone, L., Munir, T., Keatinge, M., Lambertus, M., Underhill, A., Barrett, T., Kassapis, E., Ogryzko, N.*, et al.* (2018). Dynamic control of proinflammatory cytokines Il-1beta and Tnf-alpha by macrophages in zebrafish spinal cord regeneration. Nat Commun *9*, 4670.

Vasudevan, D., Liu, Y.-C., Barrios, J.P., Wheeler, M.K., Douglass, A.D., and Dorsky, R.I. (2020). Regenerated Interneurons Integrate Into Locomotor Circuitry Following Spinal Cord Injury. BioRxiv *doi:* [*https://doi.org/10.1101/2020.03.23.003806*](https://doi.org/10.1101/2020.03.23.003806).

Wehner, D., Tsarouchas, T.M., Michael, A., Haase, C., Weidinger, G., Reimer, M.M., Becker, T., and Becker, C.G. (2017). Wnt signaling controls pro-regenerative Collagen XII in functional spinal cord regeneration in zebrafish. Nat Commun *8*, 126.

Wilkinson, R.N., Elworthy, S., Ingham, P.W., and van Eeden, F.J. (2013). A method for high-throughput PCR-based genotyping of larval zebrafish tail biopsies. BioTechniques *55*, 314-316.
